# Acute pressure overload of the right ventricle. Comparison of two models of right-left shunt. Pulmonary artery to left atrium and right atrium to left atrium: experimental study

**DOI:** 10.1186/1749-8090-6-143

**Published:** 2011-10-19

**Authors:** Mihalis Argiriou, Dimitrios Mikroulis, Timothy Sakellaridis, Vasilios Didilis, Apostolos Papalois, George Bougioukas

**Affiliations:** 1 Second Cardiac Surgery Department, Evaggelismos General Hospital, 45-47 Ipsilantou, 10676, Athens, Greece; 2 Cardiothoracic Surgery Department, Democritus University Thrace, University Hospital of Alexandroupolis, Dragana, 68100, Greece; 3 Surgical Experimental Laboratories ELPEN (AP), 95 Marathonos Avenue, 19009, Pikermi, Athens, Greece

**Keywords:** Right ventricular failure, Right ventricle overload, Pulmonary hypertension, Pulmonary artery banding, Right to left shunt

## Abstract

**Abtract:**

**Background**

In right ventricular failure (RVF), an interatrial shunt can relieve symptoms of severe pulmonary hypertension by reducing right ventricular preload and increasing systemic flow. Using a pig model to determine if a pulmonary artery - left atrium shunt (PA-LA) is better than a right atrial - left atrial shunt (RA-LA), we compared the hemodynamic effects and blood gases between the two shunts.

**Methods**

Thirty, male Large White pigs weighting in average 21.3 kg ± 0.7 (SEM) were divided into two groups (15 pigs per group): In group 1, banding of the pulmonary artery and a pulmonary artery to left atrium shunt with an 8 mm graft (PA-LA) was performed and in group 2 banding of the pulmonary artery and right atrial to left atrial shunt (RA-LA) with a similar graft was performed. Hemodynamic parameters and blood gases were measured from all cardiac chambers in 10 and 20 minutes, half and one hour interval from the baseline (30 min from the banding). Cardiac output and flow of at the left anterior descending artery was also monitored.

**Results**

In both groups, a stable RVF was generated. The PA-LA shunt compared to the RA-LA shunt has better hemodynamic performance concerning the decreased right ventricle afterload, the 4 fold higher mean pressure of the shunt, the better flow in left anterior descending artery and the decreased systemic vascular resistance. Favorable to the PA-LA shunt is also the tendency - although not statistically significant - in relation to central venous pressure, left atrial filling and cardiac output.

**Conclusion**

The PA-LA shunt can effectively reverse the catastrophic effects of acute RVF offering better hemodynamic characteristics than an interatrial shunt.

## Background

Pulmonary hypertension and right ventricular dysfunction are associated with poor survival. Management of patients with acute decompensate RV failure is largely empiric and targeted towards treating underlying precipitants while optimizing conditions of RV preload, afterload and contractility.

However, right-sided heart failure remains a major problem in the long-term follow-up, leading to impairment of functional status, severe arrhythmia, and premature death. Treatment consists of pulmonary vasodilator therapy, long-term oxygen therapy, anticoagulation, and lung transplantation, or, at times, heart-lung transplantation. Management strategies for patients who develop acute refractory right ventricular failure are:

1. Mechanical support to the failing right ventricle,

2. Conventional pulmonary vasodilators,

3. Cavopulmonary diversion in select cases, and

4. Maintenance of an adequate left ventricular performance throughout the recovery period [1].

In recent years, percutaneous balloon atrial septostomy (BAS) has been established as a palliative treatment or bridge to transplantation in patients with severe right-heart failure refractory to conventional therapy [2-5]. BAS aims at creating a "safety valve" by unloading the right heart and increasing left ventricular preload and output, peripheral perfusion, net oxygen tissue delivery, exercise tolerance, and prognosis. However, this procedure is not always successful because the size of the opening made with standard balloon septostomy techniques is imprecise and variable from patient to patient. The mortality rate is relatively high and sometimes related to severe hypoxemia from excessive right-to-left shunting through an overly large defect. Procedural mortality varies widely from 5 to 50% from single center reports. Beside this procedure has been proposed a "fontanisation" -right ventricular exclusion of the circulation- as a surgical option of RVF [6,7]. Nevertheless the presence of pulmonary hypertension is a contraindication for this procedure. Neither experimental nor clinical data are available regarding the effects of a shunt not at the atrial level but from the pulmonary artery to the left atrium. The purpose of this study was to examine the effects of right ventricle overload of two different shunts in a porcine model.

## Materials and methods

### Surgical Preparation

The animal research protocol was approved by the local authorities (A.Π. 3940/6-10-2008) in Athens. All animals used in this study were treated according to the "Guide for the care and use of Laboratory animals" published by the US National Institutes of Health (National Institutes of Health publication no. 85-23, revised 1996).

Thirty pigs weighing 22 to 35 kg were premedicated with ketamine hydrochloride (15 mg/kg IM) and midazolam (0.5 mg/kg IM), anesthetized with thiopental sodium (9 mg/kg IV bolus) and fentanyl citrate (0.5 mg IV bolus), followed by continuous IV infusions of thiopental sodium (1 mg/min), fentanyl citrate (4 mg/min), pancuronium bromide (0.25 mg/min), and lidocaine (2 mg/min), throughout the experiment. After intubation (8Ch), respiration was controlled with a Soxitronic volume respirator (Soxil S.P.A.; Segrate, Italy), supplying oxygen at 100%. No changes of tidal volume, respiratory rate, and percentage of inspired oxygen were made.

The chest was opened via a midline sternotomy, and the heart was suspended in a pericardial cradle. Catheters were placed, in the right atrium via the right external jugular vein which was surgically dissected; a right side arterial line was inserted under direct vision by a small incision in the groin, and in the left atrium directly through the left atrial appendix. To the arterial line was connected a FloTrac sensor also, (Vigileo monitor, Edwards Lifesciences) to measures parameters such as CCO, SVV/SV, SVR. This sensor is achieving measurements by pulse contour analysis based on arterial pressure waveform. In this way it is possible to avoid the use of Swan Ganz and consequently interactions with tricuspid valve function. The proximal to mid left anterior descending (LAD) coronary artery was dissected free and, a transit time flow-meter probe, (Transonic Inc. Ithaca New York 400-Series Multichannel Flowmeter) was applied. The temperature of the animal was kept within 0.5°C of the baseline value with a heating blanket and lamp. ECG, for severe rhythm disturbances, arterial pressure, central venous pressure, pulmonary artery pressure and left atrial pressure were continuously monitored. Fluid (Ringers lactate) was given at a rate of 20 ml/kg.

### Right ventricular failure model

**Figure 1:**
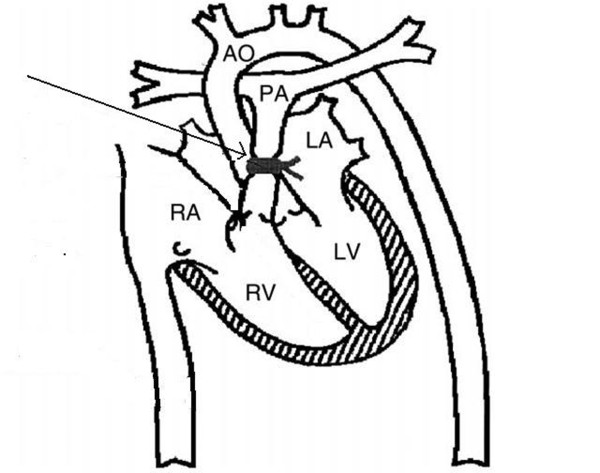
**Schematic diagram of the open-chest preparation**. Note the position of the pulmonary artery (PA) band (arrow). AO = Aorta, RA = right atrium, LA = Left Atrium, RV = Right Ventricle, LV = Left Ventricle.

To achieve RVF a banding of the very distal main pulmonary artery was performed. For banding we used a vessel loop (nylon tape) with a snare (Figure 1). The banding was persistent until pulmonary artery pressure proximal of the banding was double than pressure distally of the banding. RVF following pulmonary artery banding was defined as a profound decrease in systemic blood pressure [mean arterial pressure (MAP) < 2/3 of the beginning], an initial > 1/3 increase of systolic right ventricular pressure (RVP) and a depressed cardiac output (< 2/3 of the baseline). Additionally, right ventricular function was judged by inspection. After the completion of the banding, 30 min period was allowed for the animal to reach hemodynamic stability before the baseline recordings of pressures, CO, LAD flow and blood gazes measures.

All measurements were taken at end expiration with the ventilator turned off. Pulmonary artery band tightness was adjusted so as not to allow the systolic arterial blood pressure to fall below 60 mmHg at anytime during the experiment. With the beginning of the shunt surgery, the animals were systemically heparinized (100U/kg).

### Experimental protocol

**Figure 2:**
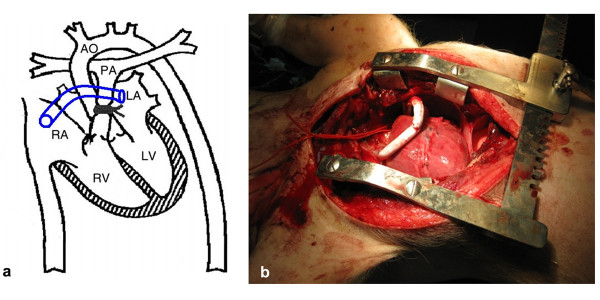
**a. Schematic diagram of the open-chest preparation with a right-left atrial shunt**. **b**. Picture of the right-left atrial shunt in the pig.

**Figure 3:**
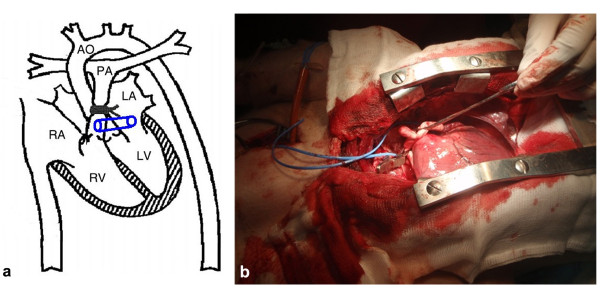
**a. Schematic diagram of the open-chest preparation with a pulmonary artery - left atrial shunt**. **b**. Picture of the pulmonary artery -left atrial shunt in the pig.

Two different settings of shunts were evaluated. Group (1) PA-LA shunt (n 15) and group (2) RA-LA shunt (n 15). A right atrial to the left atrial shunt was created with an interposition of an 8 mm PTFE graft in group No 2 (Figure 2). By means of partial vascular clamp a PTFE 8 mm graft was connected end to side with the very proximal main pulmonary artery (proximally of the banding). The other side of the graft was connected end to side with the left auriculum for group No 1 (Figure 3).

We have chosen to introduce the 14-G hypodermic needle into the left atrium, shunt, RV, pulmonary artery proximal and distal directly rather than to introduce a catheter Swan Ganz through the tricuspid valve because of the enhanced stability and reproducibility of the pressure and volumetric data from "a more complete interrogation of the RV cavity". Blood gazes samples from each cavity were selected directly from each cardiac chamber at 10 and 20 minutes from the baseline.

### Statistical Analysis

Data is expressed as mean ± standard deviation (S.D.) or median (in case of violation of normality) for continuous variables and as percentages for categorical data. The Kolmogorov - Smirnov test was utilized for normality analysis of the parameters. The comparison of variables at each time point was performed using the Independent samples t-test or the Mann-Whitney test in case of violation of normality. One factor Repeated Measures ANOVA model was used for the comparison of different time measurement of variables for each group. Pair wise multiple comparisons were performed using the method of Tukey critical difference.

To indicate the trend in the first 20 minutes of intervention, the median percentage changes after 10 and 20 minutes respectively are calculated. Comparison of percentage change from baseline of parameters during the observation period between two groups was analyzed using the Mann-Whitney test because of violation of normality.

All tests are two-sided, statistical significance was set at p < 0.05. All analyses were carried out using the statistical package SPSS ver. 16.00 (Statistical Package for the Social Sciences, SPSS Inc., Chicago, Ill., USA).

## Results

### Hemodynamics

The central venous pressure (mean), the mean pressure of left atrium, the cardiac output, the pressure of the distal portion of pulmonary artery at baseline and during 10 and 20 minutes interval were similar in both groups (Table 1).

**Table 1:** Hemodynamic measurements and statically analysis

		*baseline mean ± SD*	*10 min mean ± SD*	*20 min mean ± SD*	*% change baseline-10 min median*	*% change baseline-20 min median*
***Pulse (b/min)***	Shunt PA -LΑ	95.67 ± 10.45	112.80 ± 9.71**	105.20 ± 16.90	18.75	9.28
	
	Shunt RA -LΑ	95.67 ± 10.45	106.87 ± 18.31*	103.80 ± 13.52	8.69	9.18
	
	p-value	NS	NS	NS	NS	NS

***Arterial Blood pressure (mean)***	Shunt PA -LΑ	64.67 ± 6,72	59.33 ± 14.02 **	49.87 ± 10.08 **	-6.34	-20.31
	
	Shunt RA -LΑ	64.67 ± 6,72	59.20 ± 10.71	58.60 ± 13.43	-7.04	-14.23
	
	p-value	NS	NS	**0.054**	NS	NS

***Right Ventricular pressure (mean)***	Shunt PA -LΑ	30.00 ± 4.42	15.93 ± 4.73**	12.53 ± 4.49**	-50.0	-60.0
	
	Shunt RA -LΑ	30.00 ± 4.42	10.87 ± 3.60**	13.5 ± 5.12**	-64.0	-65.0
	
	p-value	NS	**0.022**	NS	**0.074**	NS

***Central Venous pressure (mean)***	Shunt PA -LΑ	6.93 ± 2.40	5.21 ± 2.99*	5.12 ± 3.00	-25.0	0.0
	
	Shunt RA -LΑ	6.93 ± 2.40	6.53 ± 2.72^$^	3.46 ± 3.52*	0.0	-50.0
	
	p-value	NS	NS	NS	NS	NS

***Left Atrial pressure (mean)***	Shunt PA -LΑ	5.67 ± 3.29	5.93 ± 3.51	5.93 ± 3.10	0.0	0.0
	
	Shunt RA -LΑ	5.67 ± 3.29	4.73 ± 3.03	4.47 ± 3.02	0.0	-18.0
	
	p-value	NS	NS	NS	NS	NS

***Pulmonary artery pressure (proximal)***	Shunt PA -LΑ	36.73 ± 5.28	21.73 ± 8.91**	21.63 ± 92.28**	-40.0	-40.0
	
	Shunt RA -LΑ	36.87 ± 4.70	29.03 ± 8.10**	28.90 ± 8.10**	-20.0	-21.0
	
	p-value	NS	NS	NS	NS	NS

***Pulmonary artery pressure (distal)***	Shunt PA -LΑ	12.73 ± 5.28	12.47 ± 4.81	12.40 ± 4.61	0.0	0.0
	
	Shunt RA -LΑ	17.13 ± 5.25	16.80 ± 4.83	17.27 ± 5.36	0.0	0.0
	
	p-value	NS	NS	NS	NS	NS

***Shunt pressure***	Shunt PA -LΑ		19.60 ± 4.94	19.93 ± 4.83		-5.0
	
	Shunt RA -LΑ		5.53 ± 1.40	4.87 ± 1.36		-14.3
	
	p-value		**p < 0,0005**	**p < 0,0005**		**0.023**

***CO***	Shunt PA -LΑ	4.93 ± 0.90	5.19 ± 1.22	5.39 ± 1.34	3.92	14.03
	
	Shunt RA -LΑ	4.93 ± 0.90	4.64 ± 1.02	4.87 ± 1.13	-7.69	-2.33
	
	p-value	NS	NS	NS	NS	NS

***SVR***	Shunt PA -LΑ	962.02 ± 153.04	847.17 ± 207.17*^$^	667.97 ± 207.64**	-15.44	-29.90
	
	Shunt RA -LΑ	962.02 ± 153.04	891.98 ± 221.52	815.47 ± 213.14**	-6.20	-14.81
	
	p-value	NS	NS	**0.075**	NS	**0.021**

***Flow LAD***	Shunt PA -LΑ	18.43 ± 6.83	16.14 ± 4.28	20.93 ± 7.35	-8.3	9.1
	
	Shunt RA -LΑ	18.43 ± 6.83	14.64 ± 5.37*	10.79 ± 4.98**	-28.0	-33.3
	
	p-value	NS	NS	**p < 0,0005**	NS	**p < 0,0005**

** p < 0.005 vs. baseline, * p < 0.05 vs. baseline, $ p < 0.05 vs. 20 min

There is statistically significant difference among the time measurements of heart rate variable for the PA - LA shunt, in comparison with the RA - LA shunt, especially at the 10 minute interval (p < 0.005). Pairwise comparisons show statistically significant difference between all time measurements. The heart rate variable at baseline was 95.5 ± 10.45 pulses/min, at 10 minutes interval with PA - LA shunt was 112.80 ± 9.71 pulses/min and at 20 minutes interval 105.20 ± 16.90 pulses/min, whereas with the RA - LA shunt the measurements of heart rate variable at 10 and 20 minutes interval were 106.87 ± 18.31 pulses/min and 103.80 ± 13.52 pulses/min.

There is statistically significant difference among the time measurements of mean arterial blood pressure variable for the PA - LA shunt, in comparison with the RA - LA shunt between al time measurements (p < 0.005). The mean blood pressure variable at baseline was 64.67 ± 6.72 mmHg, at 10 minutes interval with PA - LA shunt decreased at a variable of 59.33 ± 14.02 mmHg and at 20 minutes interval at 49.87 ± 10.08 mmHg, whereas with the RA - LA shunt the measurements of mean blood pressure variable at 10 and 20 minutes interval were 59.20 ± 10.71 mmHg and 58.60 ± 13.43 mmHg. Between the two groups (PA - LA shunt and RA - LA shunt) there is statistically significant difference of mean blood pressure variable at 20 min interval (p = 0,054) with the mean blood pressure of PA - LA shunt at the level of 49.87 ± 10.08 mmHg and of RA - LA shunt at the level of 58.60 ± 13.43.

As for the mean right ventricular pressure (RVP) variable, there is statistically significant difference among the time measurements of the RVP variable for the shunt PA-LΑ (p < 0.005). Pairwise comparisons show statistically significant difference between all time measurements. Also, between the two groups at 10 minute interval, a significant statistically difference (p < 0.022) is observed with the measurements to be 15.93 ± 4.73 mmHg for the shunt PA-LΑ and 10.87 ± 3.60 mmHg for the shunt RA-LΑ. Concerning the percentage change from baseline to 10 min of the mean right ventricular pressure variable, there is statistical significant difference between the two groups (p < 0.074), with 50% decrease at the PA-LA shunt and 64% decrease at the RA-LA shunt.

The variable of shunt pressure has statistically difference between the two groups at 10 minute and 20 minute interval (p < 0.005), whereas there is a significant statistically difference between groups concerning the percentage change from baseline to 20 min (p = 0.023). Comparison between all time measurements of proximal pulmonary artery pressure for both groups reveals a statistically difference (p < 0.005).

The observed decrease of SVR has a statistically difference among the 20 minute interval measurements for the shunt PA-LΑ and for the RA-LA shunt (p < 0.005). Also there is statistically difference of SVR variable between the two groups at 20 minute interval (p = 0.075) and there is statistical significant difference between the two groups concerning the percentage change from baseline to 20 minutes of the SVR variable (p = 0.021).

Another important variable that was measured was the flow at the LAD. Measurements revealed statistically significant difference among the time measurements of the LAD flow variable for the shunt RA-LΑ (p < 0.005) at 20 minute interval, with a 33.3% decrease. Between the two groups, at 20 minutes interval, the observed difference is statistically significant (p < 0.005). Finally, the observed LAD flow variable between the two groups at 20 minutes has a significant statistically difference (p < 0.005).

### Blood gases

The statistical analysis of blood gases in both groups of shunt and at all time intervals revealed no statistically difference for arterial pCO_2 _and arterial pO_2_, arterial O_2_% saturation, pulmonary artery pH, pCO_2 _of pulmonary artery and O_2_% saturation of left atrium (Table 2).

**Table 2:** Blood gases and statistically analysis.

		*baseline mean ± SD*	*10 min mean ± SD*	*20 min mean ± SD*	*% change baseline-10 min median*	*% change baseline-20 min median*
***pCO***_***2 ***_***arterial***	Shunt PA -LΑ	32.98 ± 7.61	33.23 ± 6.06	34.25 ± 6.84	0.0	5.1
	
	Shunt RA -LΑ	32.98 ± 7.61	31.28 ± 7.07	32.65 ± 6.20	-1,39	0.30
	
	p-value	NS	NS	NS	NS	NS

***pO***_***2 ***_***arterial***	Shunt PA -LΑ	377.02 ± 82.72	352.31 ± 76.01	335.65 ± 55.35*	-5.18	-8.78
	
	Shunt RA -LΑ	377.02 ± 82.72	362.27 ± 90.92	362.86 ± 90.10	0.0	0.0
	
	p-value	NS	NS	NS	NS	NS

***O***_***2***_***Sat arterial***	Shunt PA -LΑ	99.45 ± 0.94	99.24 ± 0.95	99.32 ± 0.88	-0.10	0.0
	
	Shunt RA -LΑ	99.45 ± 0.94	99.43 ± 0.90	99.59 ± 0.58	0.0	0.0
	
	p-value	NS	NS	NS	NS	NS

***pH pulmonary artery (distal)***	Shunt PA -LΑ	7.50 ± 0.09	7.44 ± 0.07	7.44 ± 0.08	-0.27	-0.66
	
	Shunt RA -LΑ	7.50 ± 0.09	7.44 ± 0.06	7.42 ± 0.07	-0.27	-0.94
	
	p-value	NS	NS	NS	NS	NS

***pCO***_***2 ***_***pulmonary artery (distal)***	Shunt PA -LΑ	36.54 ± 8.25	43.22 ± 7.19*	42.78 ± 8.26*	11.11	11.11
	
	Shunt RA -LΑ	36.54 ± 8.25	40.13 ± 8.40	41.25 ± 7.61*	4.06	10.62
	
	p-value	NS	NS	NS	NS	NS

***pO***_***2 ***_***pulmonary artery (distal)***	Shunt PA -LΑ	39.66 ± 6.40	33.40 ± 5.11**	33.19 ± 6.22**	-9.97	-14.65
	Shunt RA -LΑ	39.66 ± 6.40	33.48 ± 4.44**	35.66 ± 6.31*	-15.21	-5.47
	
	p-value	NS	NS	NS	NS	NS

***O***_***2***_***Sat pulmonary artery (distal)***	Shunt PA -LΑ	76.86 ± 9.63	65.58 ± 9.69**	64.98 ± 10.75**	-14.74	-14.28
	
	Shunt RA -LΑ	76.86 ± 9.63	64.75 ± 8.78**	65.83 ± 12.19*	-16.86	-7.39
	
	p-value	NS	NS	NS	NS	NS

***pCO2 left atrium***	Shunt PA -LΑ	36.10 ± 4.07	40.68 ± 4.31**	39.54 ± 5.15*	10.40	3.32
	
	Shunt RA -LΑ	36.10 ± 4.07	37.30 ± 4.80	35.84 ± 5.11	1.07	-6.63
	
	p-value	NS	**0.052**	**0.058**	**0.016**	**0.023**

***pO2 left atrium***	Shunt PA -LΑ	172.85 ± 43.10	99.27 ± 19.83**	118.23 ± 24.57**	-42.05	-25.50
	
	Shunt RA -LΑ	172.85 ± 43.10	114.47 ± 10.96**	121.30 ± 17.01**	-36.57	-29.94
	
	p-value	NS	**0.015**	NS	**0.050**	NS

***O***_***2***_***Sat left atrium***	Shunt PA -LΑ	99.10 ± 0.97	97.01 ± 2.20*	97.74 ± 1.88	-2.61	-1.00
	
	Shunt RA -LΑ	99.10 ± 0.97	97.46 ± 2.65	98.71 ± 0.89	-0.51	-0.31
	
	p-value	NS	NS	NS	NS	NS

** p < 0.005 vs. baseline, * p < 0.05 vs. baseline, $ p < 0.05 vs. 20 min

The decrease of pO_2 _in the pulmonary artery is statistically significant among the time measurements of the pO_2 _variable for the shunt PA-LΑ (p < 0.005). Pairwise comparisons show statistically significant difference between all time measurements. The same observations are made for the decrease of O_2_% saturation of the pulmonary artery.

pCO_2 _of the left atrium increase is statistically significant between the two groups at 10 minute interval (p = 0.052) and at 20 minute interval (p = 0.058). Τhere is also a statistical significant difference between groups concerning the percentage change from baseline to 10 minute of the pCO_2 _of the left atrium variable (p = 0.016) and the percentage change from baseline to 20 minute of the pCO_2 _of the left atrium variable (p = 0.023).

Least, the pO_2 _of the left atrium decrease reveals a statistically significant difference among the time measurements for the shunt PA-LΑ (p < 0.005) and RA-LA shunt. Pairwise comparisons show statistically significant difference between all time measurements. At 10 minute interval, between the two groups there is a statistically difference (p = 0.015), and concerning the percentage change from baseline to 10 minute, the difference between the two groups is statistical significant (p = 0.05).

It is anticipated that the minor fall of PO2 and the minor increase of PCO2 will not influence saturation because of the morphology of the oxygen-hemoglobulin dissociation curve. The discrepancy between arterial pO_2_, pCO_2 _and left atrial pO_2_, pCO_2 _can be interpreted as a technical error or as a condition error, probably because of contiguity of the sample collector to the graft.

## Discussion

Right ventricular function is identified to be an independent risk factor for mortality in various diseases as chronic obstructive pulmonary disease (COPD), pulmonary arterial hypertension (PAH) (RV failure is the end-result of PAH and the cause of at least 70% of all PAH deaths), adult respiratory distress syndrome (ARDS), etc [8]. Also pulmonary hypertension secondary to dilated cardiomyopathy constitutes a risk factor for heart transplantation procedure because of the dysfunction of the right ventricle of the graft [9]. Dysfunction of the right ventricle (RV) can occur in a number of clinical scenarios, including pressure overload, cardiomyopathies, ischemic, congenital, or valvular heart disease, arrhythmias, and sepsis. Pressure overload can occur in an acute or chronic setting [10].

Often the development of a RVF exhibits the final phase of the disease. In cardiothoracic surgery, RVF seems to be a frequent cause for postoperative cardiogenic shock associated with high mortality [11-13]. Different surgical techniques has been proposed for RVF, as atrial septostomy [3], extracorporeal right to left atrial bypass with a centrifuge blood pump and a membrane oxygenator [14], an experimental atrial septostomy with veno-venous extracorporeal membrane oxygenation (VV-ECMO) [15], or a creation of a peripheral shunt [16]. Nevertheless, the implantation of a right side assist device is associated with a high mortality [17].

**Figure 4:**
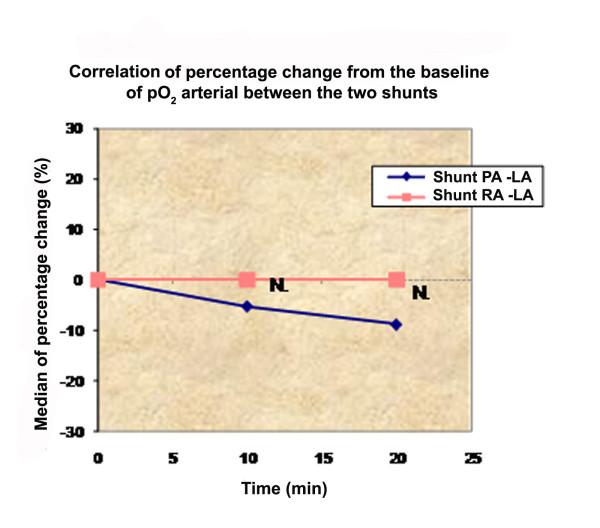
**Correlation of percentage change from the baseline of pO_2 _arterial between the two shunts**.

The first idea of a pulmonary artery to left atrium shunt was introduced 50 years ago, and belongs to Bilgutay and Lillehei [18]. Gupta evaluate in 1972 a PA-left atrium shunt in pulmonary hypertension in an experimental model [19]. The most important side effect of Gupta's model, but also in recent practice of atrial septostomy, is severe hypoxemia from excessive right-to-left shunting. Our recordings confirmed the decrease of arterial oxygen in both groups, but it was not statistical significant (Figure 4).

Besides several other mechanisms which lead to low cardiac output in RVF, a major feature is a reduced trans-pulmonary blood flow with a reduced left atrial respectively ventricular filling result, which is called serial ventricular interdependence. Our aim was to evaluate hemodynamic status of a pulmonary artery to left atrium shunt which can have many advantages and comparison of this shunt with an interatrial shunt.

Pulmonary artery banding in pigs reproducibly results in right side circulatory failure detectable as an increase in right ventricular and mean pulmonary artery pressures and a decrease in left ventricular end-diastolic pressure. In our study, in both groups after shunting it was detectable an increase in heart rate at 10 and 20 minute and a decrease of mean arterial pressure but there was statistically significant difference of mean arterial pressure between the two groups at 20 minute (p = 0.054) being more prominent in group 1 (PA-LA) shunt. This result can be explained from the concomitant decrease in this group of SVR at 20 minutes. Τhere is statistical significant difference between groups concerning the percentage change from baseline to 10 minute of the SVR variable and a statistically significant difference between the two groups at 20 minute (p = 0.075). Our recordings of a low MAP and low SVR in both groups are consistent with the results described by other investigators [20-22].

The right ventricular pressure was statistically significant higher in the group of RA-LA. Right ventricular overload - pressure lead often to life threatening ventricular tachycardias. From this point of view the PA-LA shunt has a significant advantage. We observed that right atrial pressure in both groups was not increased as expected, because the experiment was acute and the tricuspid valve by epicardial echocardiography had sufficient competence. However, an interatrial shunt is likely beneficial only if sufficient right-to-left shunting occurs to increase cardiac output.

The results of lower mean arterial pressure and SVR in favor of PA-LA shunt insinuate easier manipulation of heart function in order to optimize heart performance by simple maneuvers like volume infusion or medical intervention in cases of real conditions of right ventricle overload.

**Figure 5:**
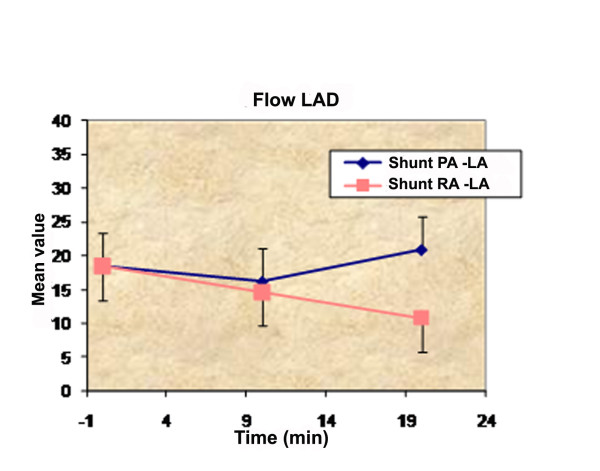
**Graphic showing the flow in the LAD and the changes during the experiment**.

**Figure 6:**
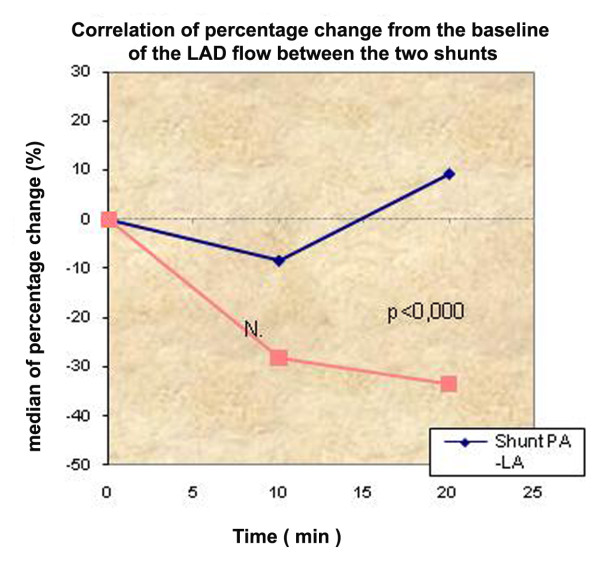
**Correlation of percentage change from the baseline of the LAD between the two shunts**.

Atrial septostomy has been associated with a risk of intraprocedural and postprocedural mortality up to 30% in several series [3,5,23-25], most commonly, secondary to progressive hypoxia, right heart failure and ventricular arrhythmias. For this reason, Zierer et al [26] had tried to determine the qualitative and quantitative impact of low-flow vs. high-flow shunting. In this study, low-flow shunting (15% of cardiac output) improved RV diastolic compliance by 42% and caused a shift of the RA reservoir-to-conduit ratio toward physiological conditions. In our study, the cardiac output was not significantly different between the two groups. This can be attributed to the Frank-Starling mechanism. According to the Frank-Starling mechanism, as the heart is stretched in response to increased preload, it augments its contraction force at the expense of increased myocardial oxygen consumption. But in our study we observed that flow in LAD had statistically significant difference between the groups concerning the percentage change from baseline to 10 minutes and statistically significant difference between the two groups at 20 minutes (p < 0.0005) in favor of the PA-LA shunt (Figure 5,6).

According the Hagen-Poiseuille law

$$Q=\frac{\pi }{8\eta }\frac{Pi-Po}{L}R^{4}$$

the PA - LA shunt has 10 fold higher volumetric flow rate, where *Q*: volumetric flow rate, *π*: mathematical constant, *η*: dynamic fluid viscosity [pascal - second (Pa·s)], *P*_*i*_: inlet pressure, *P*_*o*_: outlet pressure, *L: *total length of the tube in the *x *direction (meters), *R: *is the radius.

Because of the anatomical contiguity between pulmonary artery and left atrium, the length of the PA-LA graft is always shorter than the RA-LA graft. The pressure gradient PA-LA is always higher than the RA-LA. These two issues constitute an inherent advantage of PA-LA shunt and are rendering PA-LA shunt more effectively in that it can provide wider range of achievable flows through the shunt. Given the fact that the current technology allows the pulmonary artery banding to be adjustable, we can assume that in the future we may be able to calculate the ideal flow in an individualized manner.

To our surprise, systemic arterial de-saturation following the PA-LA shunt was not increased dramatically with devastating consequences such as systemic oxygen delivery. The advantages of a pulmonary artery to left atrium shunt are the following:

1. Can be performed without extracorporeal circulation

2. Can be used with a telemetrically controlled adjustable occlusion device, as the Flo-Watch pulmonary artery banding device (EndoArt, Lausanne, Switzerland), which has been successfully introduced in clinical practice of banding [20].

3. Can be easily occluded with the current devices, as the Gianturco-Grifka vascular occlusion device which is an appropriate closure system to occlude the shunt because of the large size (9 mm) [21]

4. Can be easily performed in conjunction with a pumpless lung assist device as Novalung in parallel with the PA shunt or in a serial setting [22].

## Conclusion

Our experiments have showed that a PA-LA shunt can more effectively moderate or even partially reverse the adverse effects of acute right ventricle pressure overload than an interatrial shunt, offering a decrease in right ventricle afterload, increased flow in left anterior descending artery with less mean arterial pressure and lower SVR.

## Limitations

Our study has some limitations. First of all, all measurements were performed in open chest surgery. Secondly, the ventilation supplying oxygen was at 100% and not at room air oxygen. Finally the measurements were taken at 10 and 20 minute interval. The above parameters may alter the results of blood gases. Nevertheless all measurements taken together allow for a realistic evaluation of the overall picture. The use of other acute RVF models and the determination of long term results are a matter of further investigations.

## Abbreviations

RV: Right Ventricle; RA: Right Atrium; RVF: Right ventricular failure; RVO: Right ventricular overload; PA-LA: pulmonary artery to left atrium shunt; RA-LA: Right atrium to left atrium shunt; LAD: left anterior descending artery; CCO: Continuous Cardiac Output; SV: Stroke Volume; SVV: Stroke Volume Variation; SVR: Systemic Vascular Resistance; COPD: chronic obstructive pulmonary disease; PAH: pulmonary arterial hypertension; ARDS: adult respiratory distress syndrome; ECG: Electrocardiogram; RVP: Right Ventricular Pressure.

## Competing interests - Disclosures

The authors declare that they have no competing interests.

## Authors' contributions

All authors read and approved the final manuscript.

MA and TS performed all the experiments, collected the data and drafted the manuscript.

AP is the clinical director of the experimental laboratory, helped out with the experiments and the data collection.

DM revised it critically for important intellectual content

VD revised it critically for important intellectual content

GB have given final approval of the version to be published
